# Correlation between bilateral lateral rectus muscle recession and myopic progression in children with intermittent exotropia

**DOI:** 10.1038/s41598-023-34441-z

**Published:** 2023-05-03

**Authors:** Jae Yong Han, Jinu Han, Sueng-Han Han

**Affiliations:** Department of Ophthalmology, Institute of Vision Research, Severance Hospital, Yonsei University College of Medicine, 50-1, Yonseiro, Seodaemun-gu, Seoul, Republic of Korea

**Keywords:** Paediatric research, Eye manifestations

## Abstract

Although several studies have reported about the relationship between the surgical correction of intermittent exotropia and myopic progression, it remains unclear, unlike the relationship between esotropia and hyperopia. Thus, this retrospective case control study evaluated the impact of bilateral lateral rectus recession in intermittent exotropia on myopic progression. This study included 388 patients with intermittent exotropia. The refractive errors and degree of exodeviation at each follow up period were analyzed. The rate of myopic progression was −0.46 ± 0.62 diopter (D)/year in patients who underwent surgery and −0.58 ± 0.78 D/year in patients who did not, with no significant difference between them (*p* = 0.254). Patients who had recurrences of more than 10 prism diopters were compared with patients who did not have. The rate of myopic progression was −0.57 ± 0.72 D/year in the recurrent group and −0.44 ± 0.61 D/year in the non-recurrent group, with no significant difference between them (*p* = 0.237). Patients with fast myopic progression had more recurrence than patients with slow progression (*p* = 0.042). Moreover, recurrence had a positive correlation with fast myopic progression (OR = 2.537, *p* = 0.021). Conclusively, the surgical correction of intermittent exotropia did not influence myopic progression.

## Introduction

Myopia is one of the major causes of refractive error resulting in visual impairment and blindness^1, 2^. Moreover, its occurrence is on the rise worldwide because of increasing indoor and near work activities. This trend is evident especially in East Asia, wherein the prevalence rate of myopia is 80–90% in a population of over 18 years^3–5^.

Many risk factors for myopia are known, including race, ethnicity, degree of education, outdoor activity, and near work^6–9^. Studies about the relationship between myopia and intermittent exotropia have been published. Ekdawi et al.^10^ performed a population-based study for 20 years and found a close relationship between intermittent exotropia and myopia. In this study, more than 90% of patients with intermittent exotropia had myopia, which was more than those in a similar population group. Some studies also reported that the increase in the vergence demand required to control exodeviation stimulates accommodation and promotes myopic progression^11–15^. Moon et al.^16^ compared the degree of myopic progression between the dominant and non-dominant eye in intermittent exotropia and found more myopic progression in the non-dominant eye. On the other hand, a 6-year retrospective observational study in school-aged children showed no significant association between intermittent exotropia and myopia^15^.

Some other studies reported the impact of surgery on myopic progression, and not just the relationship between intermittent exotropia and myopic progression. Park et al.^17^ compared both of the eyes of patients with intermittent exotropia who received unilateral lateral rectus recession. In this study, the operated eye showed faster myopic progression than the non-operated one in patients with low myopia. Meanwhile, a study reported that there was no significant difference in myopic progression between normal participants and patients who received bilateral lateral rectus muscle recession for intermittent exotropia^18^.

As mentioned above, various studies reported the relationship between myopia, intermittent exotropia, and operation for intermittent exotropia. However, unlike the relationship between esotropia and hyperopia, the relationship between exotropia and myopic progression is still not well-known. Thus, this study aimed to evaluate the myopic changes in patients with intermittent exotropia compared to the control group and investigate the correlation between bilateral lateral rectus muscle recession and myopic progression in children with intermittent exotropia.

## Results

### Demographics and baseline characteristics

A total of 388 patients with intermittent exotropia were enrolled in this study, in which 327 patients received bilateral lateral rectus recession and 61 patients did not. In the surgery group, 161 patients (49.2%) were male, and the mean age of the patients was 6.31 ± 2.02 years. The initial best corrected visual acuity (BCVA) was 0.04 ± 0.08 logarithm of the minimum angle of resolution (LogMAR). The mean amount of near deviation during the first visit was 27.17 ± 6.17 prism diopters (PD), while the far deviation was 27.02 ± 6.15 PD. The mean follow-up duration of this group was 60.47 ± 15.91 weeks. In the non-surgery group, 35 patients (57.4%) were male, and the mean age of the patients was 6.84 ± 2.23 years. The initial best corrected visual acuity was 0.03 ± 0.05 LogMAR. The mean amount of near deviation during the initial visit was 15.57 ± 6.87 PD, while the far deviation was 14.74 ± 7.41 PD. The mean follow-up duration of this group was 56.94 ± 9.79 weeks. Statistically significant differences were not observed between the two groups in terms of sex ratio, mean age, and initial BCVA. Meanwhile, significant differences were observed between the two groups in the amount of deviation at both near and far deviations (*p* < 0.001 each) and mean follow up duration (*p* = 0.023). The mean operation amount of the surgery group was 6.27 ± 1.02 mm (Table 1).

**Table 1:** Demographics and Baseline characteristics of participants.

	With surgery (n = 327)	Without surgery (n = 61)	p-value
Male:female (n)	161:166	35:26	0.266
Age at first visit (years)	6.31 ± 2.02	6.84 ± 2.23	0.074
Initial BCVA (LogMar)	0.04 ± 0.08	0.03 ± 0.05	0.449
Initial near exodeviation (PD)	27.17 ± 6.17	15.57 ± 6.87	< 0.001*
Initial distant exodeviation (PD)	27.02 ± 6.15	14.74 ± 7.41	< 0.001*
Follow up duration (weeks)	60.47 ± 15.91	56.94 ± 9.79	0.023*
Operation amount (mm)	6.27 ± 1.02		

Values were presented as mean ± SD.

*BCVA* best corrected visual acuity, *LogMar* logarithm of the minimum angle of resolution, *PD* prism diopters, *SD* standard deviation.

*Indicates statistically significant values (p < 0.05).

### Refractive errors, myopic progression, and muscle recession

The spherical equivalent (SE) of each group were compared. The initial SE (before operation) of the surgery group was −0.63 ± 1.48 diopter (D), while that of the non-surgery group was −0.86 ± 1.59 D. The surgery group’s final SE was −1.14 ± 1.67 D, while that of the non-surgery group was −1.48 ± 2.04 D. A gradual myopic shift was observed during the follow-up in both groups; the mean myopic shift was −0.49 ± 0.86 D in the surgery group and −0.62 ± 0.84 D in the non-surgery group. However, a significant difference was not observed between the two groups at all follow-up periods (*p* = 0.300 at initial visit, *p* = 0.207 at last visit). The change in SE in the two groups was also analyzed using repeated measures analysis of variance (ANOVA). To correct the differences in exodeviation between two groups, the repeated measures ANOVA was adjusted by the initial exodeviation. Both groups showed significant change over time (p < 0.001); however, a significant difference in SE change by time was not observed between them (p = 0.551). Furthermore, there was no significant association observed between exodeviation and SE change over time within each group. Post-hoc analysis using Bonferroni’s method was performed; however, a significant difference was not observed at each time. The rate of myopic progression was −0.46 ± 0.62 D/year in the surgery group and −0.58 ± 0.78 D/year in the non-surgery group. There was no significant difference between the two groups (*p* = 0.254) (Table 2). Moreover, there was no significant correlation between operation dosage and rate of myopic progression (Pearson correlation coefficient = 0.063, *p* = 0.254). We also analyzed the spherical and cylindrical change in each group during the 1-year follow-up. The mean change in sphericity in the surgery group was −0.38 ± 0.63 D, and the mean change in cylinder was −0.28 ± 0.59 D. Meanwhile, the mean change in sphericity in the non-surgery group was −0.57 ± 0.82 D, and the mean change in cylinder was −0.09 ± 0.36 D. There was no significant difference in the change in sphericity (*p* = 0.080); however, there was more cylindrical change in the surgery group (*p* = 0.002) (Table 2). In repeated measures ANOVA, each group showed significant change over time (*p* < 0.001 for both sphericity and cylinder), and a significant difference was found for cylinder between the two groups (*p* = 0.043). However, there was no significant difference for sphericity between the two groups (*p* = 0.116). Post-hoc analysis using Bonferroni’s method did not reveal any significant difference at each time between the two groups. Furthermore, within each group, there was no significant association observed between exodeviation and change in either sphericity or cylinder over time.

**Table 2:** Comparison of refractive errors and rate of myopic progression based on bilateral lateral rectus recession.

	With surgery (n = 327)	Without surgery (n = 61)	p-value
Refractive errors (D)			
Change of sphericity	−0.38 ± 0.63	−0.57 ± 0.82	0.080
Sphericity at first visit	−0.32 ± 1.39	−0.56 ± 1.75	0.314
Sphericity at last visit	−0.70 ± 1.54	−1.14 ± 2.12	0.130
Change of cylinder	−0.28 ± 0.59	−0.09 ± 0.36	0.002*
Cylinder at first visit	−0.61 ± 1.00	−0.59 ± 0.94	0.887
Cylinder at last visit	−0.89 ± 1.07	−0.69 ± 0.88	0.167
Change of SE	−0.52 ± 0.69	−0.62 ± 0.84	0.361
SE at first visit	−0.63 ± 1.48	−0.86 ± 1.59	0.300
SE at last visit	−1.14 ± 1.67	−1.48 ± 2.04	0.207
Rate of myopic progression (D/yr)	−0.46 ± 0.62	−0.58 ± 0.78	0.254

Values were presented as mean ± SD.

*SE* spherical equivalent, *D* diopters, *yr* years, *SD* standard deviation.

*Indicates statistically significant values (p < 0.05).

### Myopic progression by age group

**Figure 1:**
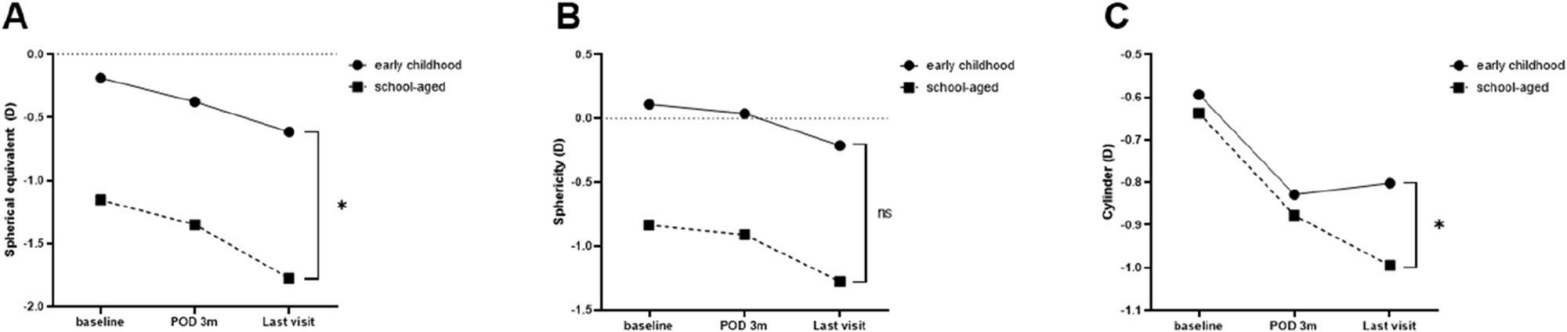
Change in refractive errors in early childhood and school-aged children. (**A**) Mean spherical equivalent showing significant myopic progression over time in each group and significant difference between both groups in the repeated measures analysis of variance (RM-ANOVA). (**B**) Mean sphericity showed significant change over time; however, there was no difference between both groups in RM-ANOVA. (**C**) Mean cylinder showing significant change over time in each group and difference between both groups in RM-ANOVA. Asterisk indicates statistically significant values (*p* < 0.05). *D* diopters, *POD* postoperative day, *m* months, *yr* year, *ns* non-specific.

The patients who underwent surgery were classified into two groups according to age: early childhood (≤ 6 years) and school-aged (> 6 years). A total of 178 patients were in the early childhood group, while 149 patients were in the school-aged group. There was no significant difference in sex ratio between the two group (p = 0.580). On the other hand, significant differences in preoperative exodeviation and operation amount were observed. The preoperative exodeviation at near was 28.01 ± 6.08 PD in the early childhood group and 26.16 ± 6.17 PD in the school-aged group (*p* = 0.007). The preoperative exodeviation at distance was 28.01 ± 6.12 PD in the early childhood group and 25.83 ± 6.00 PD in the school-aged group (*p* = 0.001). The amount of operation was significantly larger in the early childhood group (6.45 ± 0.99 mm) than in the school-aged group (6.05 ± 1.01 mm) (p < 0.001). In repeated measures ANOVA, there was significant myopic shift in SE over time in each group (*p* < 0.001), and a significant difference was observed between both groups (*p* = 0.006). However, in the case of sphericity, there was significant change over time (*p* < 0.001); however, there was no difference between both groups (*p* = 0.099). In terms of cylinder, significant change over time (*p* < 0.001) was also observed in each group, with significant difference between the two (*p* = 0.016) (Fig. 1). The rate of myopic progression between the two groups also had a significant difference. The school-aged group (−0.56 ± 0.69) showed significantly faster myopic progression than the early childhood group (−0.37 ± 0.54) (*p* = 0.007).

### Recurrence and myopic progression

The patients who underwent surgery were divided into two groups according to recurrence. A total of 39 patients (11.9%) had a recurrence, while 288 (88.1%) did not. There was no statistical difference in terms of baseline characteristics including sex ratio, mean age, initial BCVA, exodeviation before operation, and operation amount, between the two groups. Significantly more exodrift was shown in the recurrent than in the non-recurrent group, both at near (10.62 ± 3.58 vs 2.95 ± 3.97, *p* < 0.001) and distance (10.69 ± 3.52 vs 2.98 ± 4.00, *p* < 0.001). The sphericity during the baseline evaluation was −0.23 ± 0.95 D in the recurrent group and −0.33 ± 1.44 D in the non-recurrent group (*p* = 0.557). The cylinder during the baseline evaluation was −0.40 ± 1.17 D in the recurrent group and −0.64 ± 0.97 D in the non-recurrent group (*p* = 0.163). The SE during the first visit was −0.43 ± 1.33 D in the recurrent group and −0.65 ± 1.50 D in the non-recurrent group (*p* = 0.340). The change in refractive errors between the first and last visits also showed no significant difference. The rate of myopic progression was −0.57 ± 0.72 D/year in the recurrent group and −0.44 ± 0.61 D/year in the non-recurrent group, and similar to the other refractive errors mentioned above, there was no significant difference between the two group (*p* = 0.237) (Table 3).

**Table 3:** Comparison of refractive errors and rate of myopic progressions with overcorrection and recurrence.

	Recurred (n = 39)	Non-recurred (n = 288)	p-value
Change of refractive errors (D)			
Sphericity	−0.48 ± 0.75	−0.36 ± 0.61	0.356
Cylinder	−0.44 ± 1.14	−0.25 ± 0.47	0.316
Spherical equivalent	−0.70 ± 0.84	−0.49 ± 0.66	0.139
Rate of myopic progression (D/yr)	−0.57 ± 0.72	−0.44 ± 0.61	0.237

	With overcorrection (n = 46)	Without overcorrection (n = 281)	p-value
Change of refractive errors (D)			
Sphericity	−0.23 ± 0.61	−0.40 ± 0.63	0.082
Cylinder	−0.26 ± 0.38	−0.28 ± 0.62	0.853
Spherical equivalent	−0.36 ± 0.62	−0.54 ± 0.69	0.094
Rate of myopic progression (D/yr)	−0.31 ± 0.54	−0.48 ± 0.63	0.086

Values were presented as mean ± SD.

*D* diopters, *yr* year, *SD* standard deviation.

### Overcorrection and myopic progression

Patients who underwent surgery were divided into two groups according to overcorrection, in which 46 (14.1%) had overcorrection and 281 (85.9%) did not have. The mean deviation was −6.43 ± 3.44 PD at distance in a week after surgery. In the baseline characteristics, there was significant difference between the two groups except the initial BCVA (*p* = 0.066). The overcorrected group were predominantly female (*p* = 0.011) and young (*p* = 0.047). The mean amount of exodeviation was larger in the overcorrected group at all distance (*p* = 0.007 at near, *p* = 0.005 at distance). In the case of refractive errors during the baseline evaluation, a significant difference only in terms of the cylinder were observed between the two group. The sphericity during the first visit was −0.61 ± 1.22 D in the overcorrected group and −0.27 ± 1.41 D in the non-overcorrected group (*p* = 0.122). The cylinder during the first visit was −0.38 ± 0.71 D in the overcorrected group and -0.65 ± 1.04 D in the non-overcorrected group (*p* = 0.025). The SE during the first visit was −0.80 ± 1.42 D in the overcorrected group and −0.60 ± 1.49 D in the non-overcorrected group (*p* = 0.393). On the other hand, the changes in refractive errors between the first and last visits showed no significant difference. The rate of myopic progression also showed no difference (−0.31 ± 0.54 D/year vs −0.48 ± 0.63 D/year, *p* = 0.086) (Table 3).

### Factors related with fast myopic progression

Patients who underwent surgery were classified into two groups according to the rate of myopic progression per year to determine the factors related with fast myopic progression. The average rate of −1 D/year was selected as the criteria for classification based on the average threshold used for myopia treatment^19, 20^. A total of 54 (16.5%) patients had fast progression, while 273 (83.5%) patients had slow progression. The fast progression group underwent significantly smaller amounts of surgery (*p* = 0.011), had smaller amount of exodeviation (*p* = 0.039 at near, *p* = 0.014 at distance), showed more recurrence rate (*p* = 0.042) and myopic sphericity during the baseline visit (*p* = 0.037) (Table 4.). Furthermore, the factors associated with fast myopic progression were analyzed using multivariable regression analysis. Sphericity during the first visit (OR = 0.776, *p* = 0.017) and operation amount (OR = 0.676, *p* = 0.019) showed negative association, and recurrence (OR = 2.537, *p* = 0.021) showed positive association with myopic progression (Table 5).

**Table 4:** Comparison of baseline characteristics in the fast and slow progression group.

	Fast progression (n = 54)	Slow progression (n = 273)	p-value
Baseline characteristics			
Male:female (n)	24:30	137:136	0.460
Age at operation (years)	6.59 ± 1.51	6.25 ± 2.10	0.163
Operation amount (mm)	5.98 ± 0.85	6.32 ± 1.04	0.011*
Initial near exodeviation (PD)	25.83 ± 4.83	27.43 ± 6.38	0.039*
Initial distant exodeviation (PD)	25.37 ± 5.03	27.34 ± 6.31	0.014*
Overcorrection after surgery, n, %	5 (9.3)	41 (15.0)	0.296
Recurrence after surgery, n, %	11 (20.4)	28 (10.3)	0.042*
Refractive errors at first visit (D)			
Sphericity	−0.72 ± 1.52	−0.24 ± 1.35	0.037*
Cylinder	−0.66 ± 1.20	−0.61 ± 0.96	0.727
SE	−1.05 ± 1.74	−0.55 ± 1.42	0.051

Values were presented as mean ± SD.

*n* numbers, *PD* prism diopters, *D* diopters, *SE* spherical equivalent, *SD* standard deviation.

*Indicates statistically significant values (p < 0.05).

**Table 5:** Factors associated with fast myopic progression in patients who underwent bilateral lateral rectus recession.

	Univariate analysis			Multivariable analysis			VIF
	OR	95% CI	p-value	OR	95% CI	p-value	
Age at operation	1.086	0.941–1.254	0.258				
Sphericity at first visit	0.792	0.647–0.969	0.023*	0.776	0.629–0.956	0.017*	9.121
SE at first visit	0.815	0.680–0.976	0.026*				
Operation amount	0.697	0.509–0.955	0.025*	0.676	0.488–0.937	0.019*	17.095
Initial near exodeviation	0.955	0.906–1.006	0.084				
Initial distant exodeviation	0.942	0.892–0.995	0.033*				
Overcorrection	0.5577	0.217–1.536	0.271				
Recurrence	2.238	1.037–4.830	0.040*	2.537	1.150–5.595	0.021*	1.049

*OR* odds ratio, *CI* confidence interval, *VFI* variance inflation factor.

*Indicates statistically significant values (p < 0.05).

## Discussion

The patients in this study showed significant myopic progression during follow-up regardless of surgical correction, which is in accordance with previous studies. Ekdwai et al.^10^ reported a relationship between intermittent exotropia and myopic progression, in which 91.1% patients with intermittent exotropia showed myopia during the 20-year follow-up. Moreover, Shin et al.^18^ also reported a significant myopic progression in 210 early school-aged children.

In this study, there was no significant difference in myopic progression according to bilateral lateral rectus recession. Some previous studies reported similar results to this study. Ekdwai et al.^10^ compared the myopic progression of 54 patients with intermittent exotropia who underwent bilateral rectus recession and 81 patients who did not. In this report, there was no significant difference between them. Shin et al.^18^ also compared the myopic progression in 86 patients who underwent surgery and 54 patients who did not and reported that there was no significant difference between them. On the other hand, Park et al.^17^ reported different results. They compared both eyes of patients who received unilateral lateral rectus recession. In this retrospective report, the low myopia group showed significantly more rapid myopic progression in the operated eyes than in the un-operated ones. However, similar to our results, there was no significant difference in patients with refractive errors other than low myopia.

On the other hand, patients who underwent surgery showed significantly more cylindrical change than patients who did not undergo surgery. These results are similar to those of previous studies that reported a significant increase in cylinder after receiving surgery^17, 21–23^. A little difference was the shorter duration of cylindrical change. Those studies showed shorter than 6 months of cylindrical change; however, it was maintained for almost 1 year in this study.

Our study also performed additional analyses on patients who underwent surgery. At first, we compared the myopic progression of patients aged around 6 years. In our study, there was a significant difference in the rate of myopic progression between early childhood and school-aged children. This was superficially in accordance to a previous study by Tricard et al.^24^, wherein they reported that 7–9 and 10–12 year-old children showed faster progression. However, we also found different results through a more detailed analysis. The components of SE, sphericity, and cylinder showed different results. Unlike SE, sphericity was not different between the two groups. The two results are contradictory in terms of myopic progression, and this is because the change in SE was influenced by the cylinder. The cylinder had decreased during the last visit in the early childhood group, which might have increased the gap in SE between the two groups. The reason might be the decrease in astigmatism over years due to the emmetropization of children^25^. At the same time, because the operation amount of patients in the early childhood group was significantly larger, surgery-induced astigmatism should have been increased more than in the school-aged group. Hence, further evaluation about the cylindrical change by age is needed.

Second, patients who experienced a recurrence of more than 10 PD after surgery were compared with patients who did not have recurrence, and there was no significant difference in myopic progression. Based on previous studies about accommodation and myopic progression^12, 13, 26^, the increased demand for accommodative convergence after recurrence might have caused more myopic progression than in patients who showed orthotropia after surgery. However, our study showed no significant difference, which is in accordance with a recent report that showed skepticism about the relevance of accommodation and myopic progression^27^. However, in terms of progression rate, the group with faster rate had significantly more recurrence after surgery. In multivariable analysis, recurrence showed significant positive association (Table 5). These results partially conflict with each other. However, as shown in Table 3, the rate of myopic progression was faster in the recurrent group, although it was not significant. Thus, it may be difficult to state that there is no correlation between myopic progression and recurrence. For clearer results, further evaluation is needed.

Nevertheless, this study still has some limitations. First, this was a retrospective study; hence, there was little difference in the last follow-up period among the patients. Second, the refractive errors in all patients were measured only using manifest refraction. In general, the gold standard for refractive measurement in children is cycloplegic refraction. Moreover, previous studies reported some difference between cycloplegic and non-cycloplegic refraction. This difference is especially severe in patients with hyperopia and emmetropia; however, in the case of patients with myopia, the difference was smaller: non-cycloplegic refraction shows 0.09–0.25 D more myopia^18, 28–30^. The more important point is that this study aimed to confirm changes in the amount of myopia. Since the entire refraction test was conducted without cycloplegia in this study, there would be no problem with the reliability of the results. Third, although the total number of participants in this study was larger than that of previously published studies, the number of patients who did not undergo surgery was relatively smaller compared to those who received surgery. Fourth, other factors that may affect the progression of myopia—outdoor activities, degree of near work, and family history—were not reflected in the study. One example of is axial length, which has been shown to have a high correlation with myopia in many studies. However, due to the retrospective design of our study, axial length was not included for analysis. If future research includes an analysis of both refractive error and biometric data such as axial length, the correlation would be more accurate. Lastly, since the purpose of this study was to confirm the effect of surgery, one disadvantage is that the entire follow-up period was relatively shorter than others. However, if the observation period was set for a longer time, other environmental factors besides surgery would have greater impact, and the reliability of the results would be further reduced.

Our study results indicate that bilateral lateral rectus recession in patients with intermittent exotropia do not affect myopic progression. Moreover, there was no significant change in myopic progression by recurrence and overcorrection after bilateral lateral rectus recession. However, careful follow-up is still needed because rapid myopic progression can be expected in patients with recurrence.

## Methods

The protocol of this study was approved by the institutional review board of Severance Hospital, Yonsei University College of medicine, Korea(YUHS-SH-IRB-4-2022-1332). Because of the retrospective design of the study, informed consents were waived according to the institutional review board. The study was performed in accordance with the tenets of the Declaration of Helsinki.

The medical records of patients who were younger than 13 years and diagnosed with intermittent exotropia between March 2016 and June 2021 were retrospectively reviewed. Patients who received nearly 1 year of follow-up were included. All patients received manifest refraction and alternative cover test at each visit, and patients with poor cooperation during the evaluation were excluded. Patients with amblyopia, history of previous ocular surgery including reoperation due to recurrent strabismus, previous history of treatment for myopic progression, and any other ocular and neurologic disease associated with myopic progression were also excluded.

The patients were classified into two groups: patients who received bilateral lateral rectus muscle recession and patients who did not. The patients with surgery visited the clinic prior to surgery (up to 1 months) and 1 week, 3 months after surgery. Last visit of surgery group was about a year after surgery. The patients without surgery visited the clinic at baseline and a year after the first visit.

The patients’ demographics including gender, age, refractive errors, and deviation angle of exotropia were collected. Deviation was measured using the alternative prism cover technique and performed at both near (33 cm) and distance (6 m). Measurement was performed under full correction of refractive errors. Refraction was done through manifest refraction using retinoscopy. In the surgery group, refraction was measured preoperatively, at 3 months and the last visit. In the non-surgery group, refraction was measured during the first and last visits.

The rate of myopic progression was calculated using the mean spherical equivalent (SE) change per year. In this study, the mean SE between both eyes were highly correlated (Spearman correlation coefficient = 0.865, *p* < 0.001); therefore, only the measurement from the right eye of each patient was analyzed.

Additional analyses were performed in patients who underwent surgery. They were grouped according to age, recurrence, overcorrection, and degree of myopic progression. First, patients were divided into two groups. Those who were under 6 years of age were in the early childhood group, and those who were 7 years or older were in the school-aged group. Recurrence was defined as an exodeviation of more than 10 PD after the operation at 1 year follow up period. If a patient’s exodeviation was corrected more than what was previously predicted (showed esodeviation postoperatively), and remained still at 1 month follow up, the patient was classified under the overcorrected group. Furthermore, rate of -1 D/year in SE was selected as a standard for determining fast myopic progression. This figure was selected based on the average threshold used for myopia treatment^19, 20^.

Statistical analyses were performed using IBM SPSS for Windows, version 26.0 (IBM Corp., Armonk, NY, USA). P-value < 0.05 was considered to be statistically significant. The paired t-test, independent t-test, chi-square test, Fisher’s exact test, Mann–Whitney test, and repeated measures analysis of variance (ANOVA) were performed for statistical comparison. Univariate analysis was performed for each variable to determine the factors related to faster myopic progression. Variables with p-value < 0.3 in univariate analysis were included in the multivariable regression analysis (Logistic regression model). The odds ratios with 95% confidence intervals were calculated.

## Data Availability

The datasets generated and/or analysed during the current study are not publicly available due to the restriction of the Institute of Institutional Review Board of Severance Hospital, Yonsei University College of medicine, Korea. But are available from the corresponding author on reasonable request.
